# Programmed Cell Death Protein Ligand 2 Is a Potential Biomarker That Predicts the Efficacy of Immunotherapy

**DOI:** 10.1155/2021/9453692

**Published:** 2021-10-31

**Authors:** Aoyun Wang, Han Chu, Zheng Jin, Qingzhu Jia, Bo Zhu

**Affiliations:** ^1^Institute of Cancer, Xinqiao Hospital, Third Military Medical University, Chongqing 400037, China; ^2^Chongqing Key Laboratory of Tumor Immunotherapy, Chongqing 400037, China; ^3^Center of Growth, Metabolism and Aging, Key Laboratory of Bio-Resources and Eco-Environment, College of Life Sciences, Sichuan University, Chengdu 610064, China; ^4^Research Institute, GloriousMed Clinical Laboratory (Shanghai) Co., Ltd., Shanghai 201318, China

## Abstract

Immune checkpoint inhibitor (ICI) responses vary, and biomarkers for predicting responders are urgently needed. Growing evidence points to the association between programmed cell death protein ligand 2 (PDL2) and ICI benefits, while clinical evidences were lacking. Thus, we consolidated five public ICI-treated cohorts to investigate the association between PDL2 expression and ICI treatment prognosis. Immune cell signatures and IFN-*γ* signatures are investigated in The Cancer Genome Atlas (TCGA) dataset and later in ICI-treated cohorts to explore the association between PDL2 and antitumor immunity in the tumor microenvironment (TME). We found that immune cell signatures and IFN-*γ* signatures were enriched in the PDL2-high group in TCGA pooled cohorts and most cancers. Consistently, in ICI-treated cohorts, patients with high PDL2 expression experienced longer overall survival time (OS) and were more likely responsive to ICIs than patients with low PDL2 expression. Immune cell scores of the high PDL2 expression patients were significantly higher (*P* < 0.05) than those of the low PDL2 expression patients in ICI-treated cohorts. In conclusion, our findings suggest that PDL2 is a potential predictive biomarker for ICIs.

## 1. Introduction

ICIs, which combine the proteins of programmed cell death protein 1 (PD-1) axis or cytotoxic T lymphocyte-associated protein 4 (CTLA-4), are capable of improving the clinical prognosis of patients with various forms of cancers [1–4]. However, the low response rate in unselected populations limits their clinical efficacy. The active PD-1 signature in the tumor microenvironment is attributable for the escape of tumor immunity [5, 6]. Binding of PD-1 to its ligands, programmed cell death protein ligand 1 (PDL1) and PDL2, can result in a failed CD8+ T cell activation, which weakens the signals produced by CD28 and T cell receptor [7, 8]. PD-1 is mainly expressed by activated T cells, and PDL1 appears on antigen-presenting cells (APCs) or tumor cells [9]. Thus, PDL1 immunohistochemistry (IHC) is now the mainstay for the clinical screening of ICI responders from nonresponders. However, in previous studies, the responses to ICI treatment were inconsistent; some PDL1-positive patients responded poorly, while some PDL1-negative patients had considerable response rates [10–12]. Even in many clinical trials, no correlation was observed between the PDL1 expression and ICI treatment benefits [13–15]. Therefore, new predictive biomarkers are urgently needed for patients with cancer.

PDL2 is the other ligand for PD-1. Unlike PDL1, PDL2 was initially only observed in APCs [6]. Recently, after microenvironmental stimulation, researchers found that many immune cells and tumor cells could express PDL2 [16–19]. PDL2 is also considered a potential therapeutic target in prostate cancer [20]. One clinical study showed incomplete expression between the PDL1 and PDL2 in head and neck squamous cell carcinoma patients [21]. Consistently, animal models indicated combined anti-PDL1 and anti-PDL2 could abolish the tolerance after single anti-PDL1 treatment [22, 23]. Vivo trails show PDL1-specific and PDL2-specific T cells represent different T cell antigens [24]. However, the investigation between PDL2 and the prognosis of patients across multiple cancers receiving ICI treatment was still lacking, and the possible behavior of PDL2 in the TME remains unclear. Therefore, we conducted this study using data collected from RNA-seq and clinical information of published ICI cohorts to explore the effect of PDL2 expression on the prognosis of patients receiving ICI treatment for different cancers. We also used TCGA database to explore the characteristics of PDL2 in the TME.

## 2. Materials and Methods

### 2.1. Public Data Collection

The mRNA expression profiles of TCGA patients were downloaded from the Genomic Data Commons (https://portal.gdc.cancer.gov/) using the R package TCGAbiolinks (https://bioconductor.org/packages/release/bioc/html/TCGAbiolinks.html). The samples that had sequencing quality evaluated as “A” were retained. GRCh38.homo (https://asia.ensembl.org/index.html) was used for Gene ID conversion; the mRNA data of non-protein-coding genes were excluded. Considering that LAML is not a solid tumor, patients with LAML were excluded. In each type of cancer, we divided samples into two groups using the median of PDL2 expression.

Three melanoma cohorts (Gide cohort: *n* = 73; Liu cohort: *n* = 121; and Van Allen cohort: *n* = 41), a bladder cancer cohort (Mariathasan cohort: *n* = 348), and a clear cell renal cell carcinoma cohort (Miao cohort: *n* = 33) with detailed clinical information, response data, and mRNA-seq data from published researches were analyzed [25–29]. All patients were treated with anti-PD-(L)1, anti-CTLA-4, or anti-PD-(L)1 combined with anti-CTLA-4. The efficacy of antitumor immunotherapy was evaluated by the Response Evaluation Criteria in Solid Tumors (RECIST) version 1.1. Patients who achieved complete response (CR) and partial response (PR) or had stable disease (SD) that lasted for >6 months were considered to have response to ICI treatment. All other patients were considered to have no response to ICI treatment.

### 2.2. Gene Set Enrichment Analysis

We performed GSEA (https://www.gsea-msigdb.org/gsea/index.jsp) to detect the distribution of immune genes in TCGA cohorts. CD8+ T cell signature, DC signature, Th1 signature, and IFN-*γ* gene signatures were collected from published studies [30–33]. The immune infiltration scores of three cell types in each ICI sample were calculated using Single Sample Gene Set Enrichment analysis (ssGSEA) of GSVA of R package (http://bioconductor.org/packages/release/bioc/html/GSVA.html). The fragments per kilobase of transcript per million mapped reads (FPKM) of protein-coding genes between the high PDL2 expression and low PDL2 expression groups were analyzed in each cancer and TCGA pooled cohort. The results were considered statistically different when normalized *P* values (*P*) were <0.05.

### 2.3. Statistical Analysis

Univariate Cox regression was used to analyze whether high PDL2 expression is protective (0 < hazard ratio (HR) < 1) or increases the risk (HR > 1) regarding the prognosis of ICI treatment. The overall survival (OS) was assessed and compared between different groups using Kaplan-Meier (KM) method and a log-rank test. Wilcoxon's signed rank test was used to compare the difference of infiltration scores between the PDL2-high group and the PDL2-low group in each ICI cohort. *P* value of <0.05 was considered to indicate statistical significance in these analyses. All statistical analyses were performed on R version 4.0.0.

## 3. Results

### 3.1. PDL2 Is Associated with an Activated Antitumor Environment in TCGA

**Figure 1:**
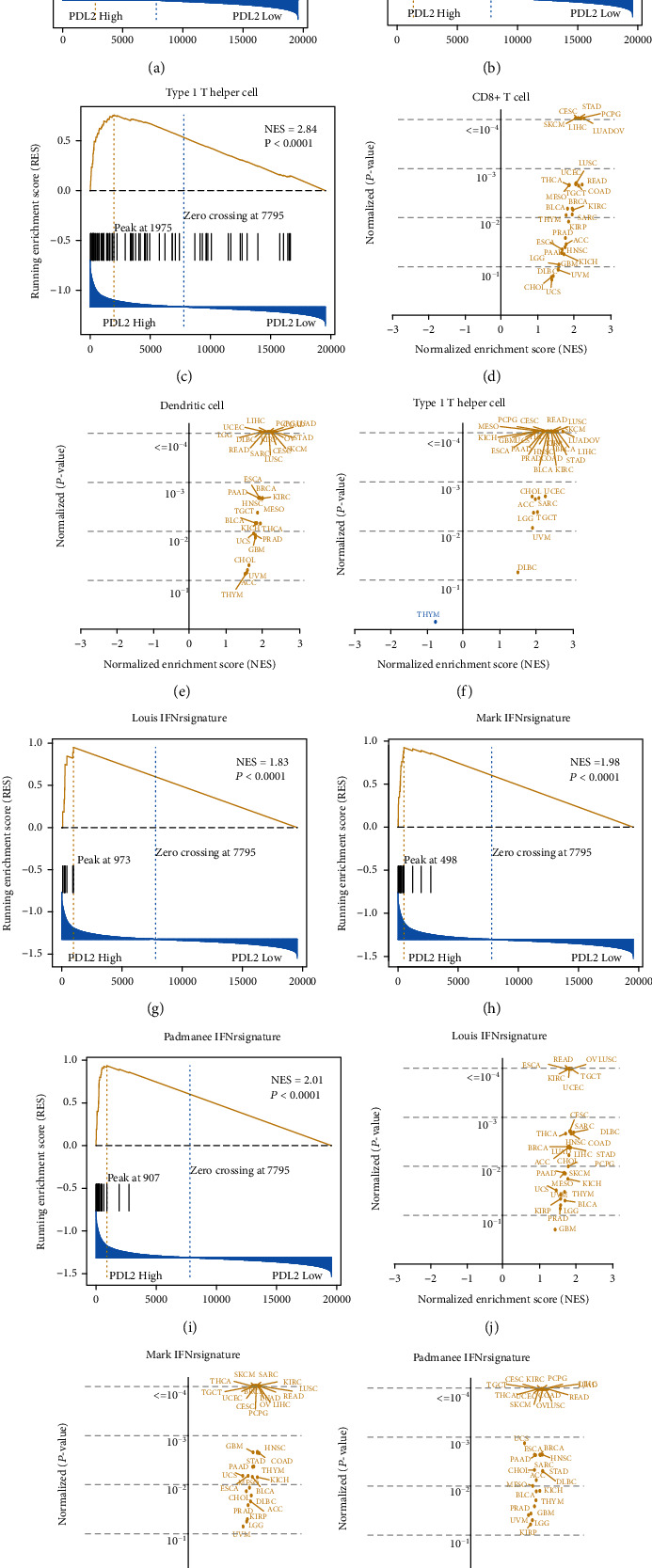
Enrichment and summary plots of the immune-related signatures in TCGA. Enrichment plots of (a) CD8+ cell signature, (b) dendritic cell signature, and (c) Th1 signature in TCGA pooled cohort, all cell signatures of the PDL2-high group were mostly enriched. (d, e) Summary plots of cell signatures with enrichment NES and *P* values in each tumor type, except for the Th1 signature enrichment in the PDL2-low group of THYM, all results are consistent with the results of TCGA pooled cohort. (g–i) Enrichment plots with IFN-*γ* signatures in TCGA pooled cohort, all IFN-*γ* signatures were enriched in the PDL2-high group. (j–l) Summary plots of IFN-*γ* signatures with enrichment NES and *P* value in each tumor type, all IFN-*γ* signatures were enriched in the PDL2-high group.

To investigate the possible effect of PDL2 on immune cell infiltration, GSEA was performed in TCGA pooled cohort. As shown in Figures 1(a)–1(c), CD8+ T cell signatures (NES = 1.82, *P* = 0.0083), DC signature (NES = 2.44, *P* < 0.0001), and Th1 signature (NES = 2.77, *P* < 0.0001) were significantly enriched in the high PDL2 expression group. To evaluate the differences between different tumor types, we performed GSEA in 32 separate cancers (Figures S1–S3). Although NES was slightly different, CD8+ T cell signature and DC signature were enriched in the PDL2-high group in most cancer types (Figures 1(d) and 1(e)). Th1 cell signatures of most cancers were enriched in the PDL2-high group, but in the THYM group, the PDL2-low group showed more intense Th1 cell signatures than the PDL2-high group. Generally, PDL2 expression is associated with CD8+ T cell, DC, and Th1 cell infiltration, which critically operate in cancer immunotherapy.

Suppressed T cell activation is crucial to the tumor immune escape mechanisms; we collected three IFN-*γ* signatures to assess the level of T cell activation: Louis signature (NES = 1.85, *P* < 0.0001), Mark signature (NES = 2.07, *P* < 0.0001), and Padmanee signature (NES = 2.10, *P* < 0.0001), which were significantly enriched in the PDL2-high group, suggesting that stronger T cell activation may exist in patients of the PDL2-high group (Figures 1(g)–1(i)). Individual analysis was performed (Figures S4–S6). Three IFN-*γ* signatures tend to be enriched in the PDL2-high group among cancers (Figures 1(g)–1(i)): DLBC, LUSC, COAD, SARC, and TGCT were the leading cancers with the highest NES by the Louis signature analysis (Figure 1(j)). By the Mark signature and Padmanee signature analysis, LUSC, READ, LUAD, HCC, and COAD were detected as the top 5 NES rankings, but Mark signature rankings were LUSC, COAD, LUAD, READ, and HCC; Padmanee signature ranking was READ, LUSC, HCC, LUAD, and COAD (Figures 1(k) and 1(l)). Generally, the PDL2-high group showed a more intense IFN-*γ* signature than the PDL2-low group. Better antigen presentation, T cell activation, and stronger cytotoxicity may exist in the PDL2-high group.

### 3.2. Association of PDL2 Expression with Prognosis of ICI Treatment

**Figure 2:**
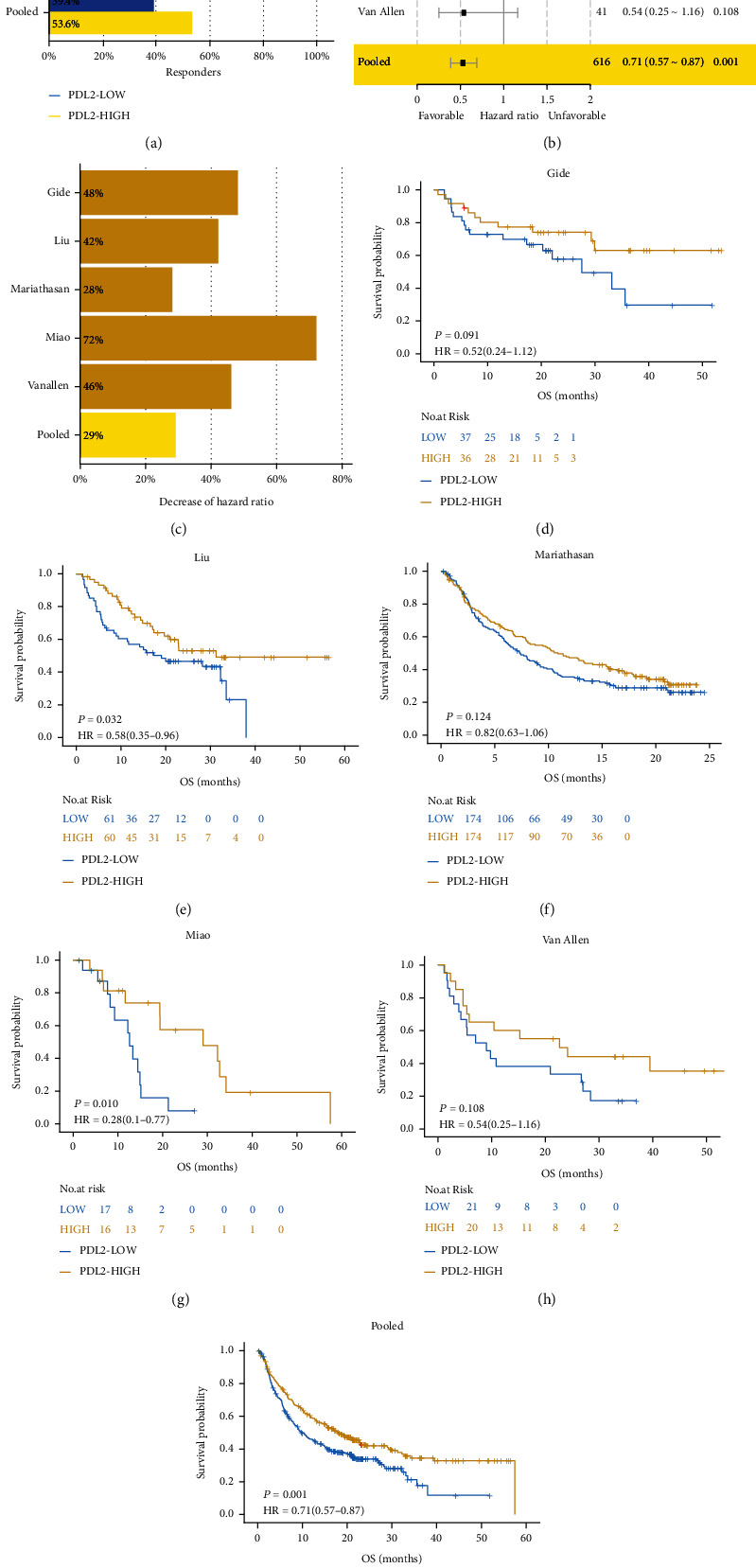
Survival analysis between the PDL2-high group and the PDL2-low group in the ICI cohorts. (a) Histogram describing proportions of responders in different groups of ICI cohorts; except the Miao cohort and Van Allen cohort, the PDL2-high group has more responders than the PDL2-low group. (b) Univariate Cox analysis according to PDL2 expression median in the ICI cohorts; high PDL2 expression is protective in ICI-treated patients. (c) The percentage decrease of HR caused by high PDL2 expression in the ICI cohort. (d–i) KM plot of OS in ICI cohorts comparing patients with high and low PDL2 expressions and longer OS were observed in the PDL2-high group.

Since PDL2 was related to the antitumor immune environment, we first aimed to determine whether PDL2 affects the prognosis of ICI treatment. Higher response rate of the PDL2-high group was observed in the Gide cohort, Liu cohort, Mariathasan cohort, and pooled cohort (Figure 2(a)). In the pooled cohort, the response rate of the PDL2-high group was 53.6% compared to 39.4% in the PDL2-low group (Figure 2(a)). Consistent with the result of response rate, high expression of PDL2 is a positive factor for survival in the ICI cohort (Figures 2(b) and 2(c)). The patients in the PDL2-high group experienced longer OS than those in the PDL2-low group when analyzing in the Liu cohort, Miao cohort, and pooled cohort (Figures 2(e), 2(g), and 2(i); Liu: HR [95%CI] = 0.58 [0.35–0.96], *P* = 0.032; Miao: HR [95%CI] = 0.28 [0.10–0.77], *P* = 0.010; pooled: HR [95%CI] = 0.71 [0.57–0.87], *P* = 0.001). Longer OS was also seen in patients in the PDL2-high group in the Gide cohort, Mariathasan cohort, and Van Allen cohort; however, those results were not statistically significant (Figures 2(d), 2(f), and 2(h); Gide: HR [95%CI] = 0.52 [0.24–1.12], *P* = 0.091; Mariathasan: HR [95%CI] = [0.63–1.06], *P* = 0.124; Van Allen: HR [95%CI] = [0.25–1.16], *P* = 0.108).

We next investigated the effect of PDL2 expression on non-ICI treatment prognosis in TCGA database. We found that there is no significant difference in OS between the PDL2-high group and the PDL2-low group in TCGA pooled cohort (Figure S7, HR [95%CI] = 0.98 [0.90–1.07], *P* = 0.603), suggesting that higher PDL2 expression was not a prognostic factor in non-ICI treatment.

### 3.3. Verifying Association between PDL2 and TME in ICI Cohorts

**Figure 3:**
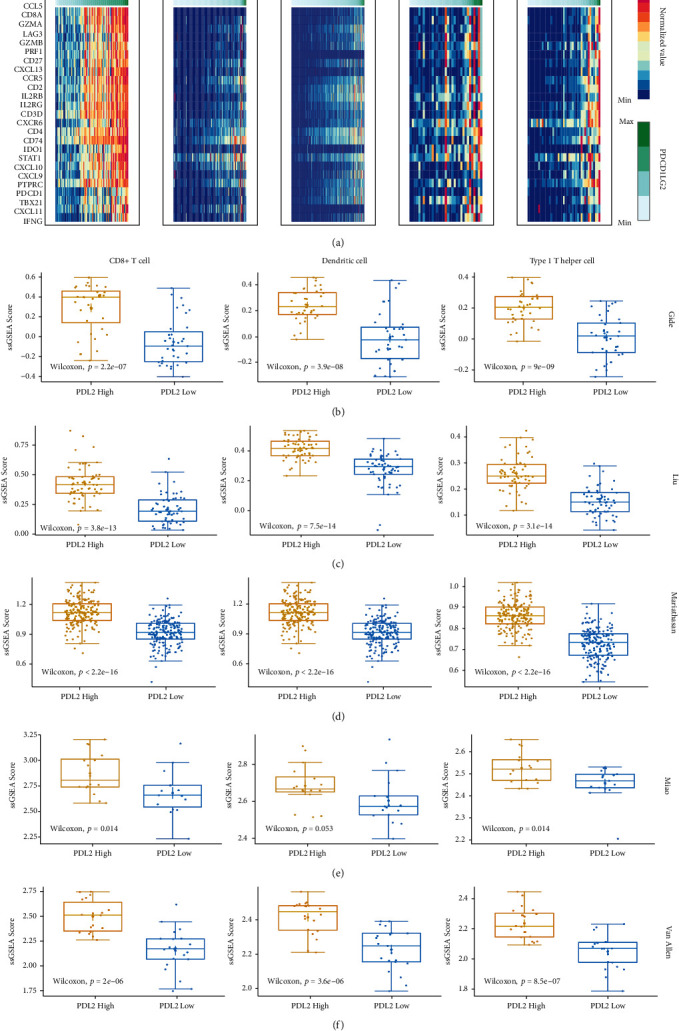
Association between PDL2 expression and immune-related signatures in the ICI cohorts. (a) Heat map of Padmanee IFN-*γ* signature genes in ICI-treated cohorts. (b–f) The ssGSEA score of immune cell signatures comparing between the PDL2-high group and the PDL2-low group in the ICI-treated cohorts. Wilcoxon's test was used to compare the differences.

We explored the relationship between PDL2 and TME in the ICI-treated cohorts. We first evaluated the expression of Padmanee IFN-*γ* immune genes and PDL2 (Figure 3(a)). Thereafter, the immune cell infiltration of each sample was quantified by ssGSEA in the ICI cohorts. In the Gide cohort, the ssGSEA scores of CD8+ T cell, DC, and Th1 cells were higher in the PDL2-high group than the scores in the PDL2-low group (Figure 3(b)), and this is consistent with the results of the analysis of other melanoma cohorts (Figures 3(c) and 3(f)). In bladder cancer and the Mariathasan cohort, higher ssGSEA scores were observed in the PDL2-high group (Figure 3(d)). In the Miao cohort, T cell infiltration and Th1 cell infiltration were still significantly higher in the PDL2-high group; the DC infiltration trends were also higher in the PDL2-high group, but not significantly (Figure 3(e)). These results suggest that high expression of PDL2 is accompanied by an antitumor immune environment in the ICI cohorts.

## 4. Discussion

This study investigated the potential use of PDL2 expression as a prognostic marker of the effect of ICI treatment by integrating published RNA-seq with clinical data. Our study indicated that high expression of PDL2 is associated with high rates of response to treatment by ICIs and long OS for patients across multiple cancer types. The immune cell analysis indicated that higher expression of PDL2 may be accompanied with higher level of CD8+ T cell, DC, and Th1 cell infiltration; additionally, IFN-r signature analysis suggests better T cell activation may occur in patients with high PDL2 expression. Our study is one of the first to propose PDL2 as a predictive biomarker for analyzing responses to ICI treatment across multiple cancers.

CD8+ T cells directly participate in the killing of tumor cells in TME [34]. PDL1 is expressed by tumor cells to resist the antitumor immunity mediated by CD8+ T cells, which prevents T cell activation [9]. This expression is induced by the mutations of tumor cells and the IFN-*γ* secreted by T cells [35]. IFN-*γ* from T cells is a major factor for most cancers [36]. PDL1 expression usually reflects the high IFN-*γ* secreted in the TME, which indicates the strong antitumor immunity, such as CD8+ T cell response. Thus, PDL1 is a predictive marker of ICI treatment. However, in some clinical experiments, high PDL1 expression was not associated with the clinical effect of ICI treatment, and PDL1-negative patients benefited from ICI treatment, suggesting that PDL1 cannot fully represent the antitumor immunity [10–12]. PDL2 is another ligand that received less research attention in the early stages. Recent evidence proves that PDL2 is closely associated with the TME [23]. The IHC results of PDL2 in different cancers were inconsistent with the IHC results of PDL1, in which PDL2-positive IHC and PDL1-negative IHC patients responded to the ICI treatment [21]. Animal models showed overexpression of PDL2, which can induce a rapid tumor proliferation, and anti-PDL2 treatment could eradicate the resistance to anti-PDL1 drugs [23]. Our results are in agreement with the above research, and we found that high expression of PDL2 is associated with an activated antitumor environment in both TCGA database and the IO cohorts. The expression of PDL2 could be an evidence of ICI treatment.

In previous research, the expression of PDL2 was induced by the NF-*κ*B pathway and the signal transducer and activator of transcription- (STAT-) 6 pathway [37]. Knockout NF-*κ*B cannot eliminate PDL2 expression [38]. After IFN-*γ* and LPS stimulation, DCs from NF-*κ*B p50^−/−^p65^−/+^ mice were incapable of upregulating PDL2 expression [38]. However, another study showed that NF-*κ*B p50^−/−^ mice cannot produce IL-4 and IL-13, the activators of STAT-6 [39]. STAT-6 is an important transcription factor and signal transduction molecule for many biological processes, especially for the Th2 immune responses [40]. As it is induced by NF-*κ*B and STAT-6, PDL2 may be involved in the Th2 immune response [37]. However, in our research, PDL2 expression was strongly correlated with Th1 cell infiltration which may be because PDL2 is coexpressed with PDL1. This was investigated to reveal that PDL2 participates in the Th1 immune response. However, more investigation is needed to clarify how PDL2 affects the TME.

This research has some limitations, the limited sample size in one ICI-treated cohort that may have introduced statistical bias. However, consistent results from numerous cohorts may have minimized the bias. Secondly, the availability of limited information prevented us from verifying the influences of functional PDL2 mutation on the prognosis of ICI treatment. Considering that the IHC results of PDL2 could provide a clear cut-off value, an investigation on the PDL2 mutation may clarify the immune functions of PDL2. More molecular studies involving animal models and cell lines are needed. Thirdly, the objective response rate (ORR) of many ICI-treated patients was not evaluated, which may have weakened the statistical analysis; however, the pooled cohort analysis has minimized such biases.

## 5. Conclusions

In summary, our results indicated that PDL2 was associated with an antitumor effect and can be a potential predictive biomarker to screen patients' benefit from ICI treatment in multiple cancers.

## Data Availability

TCGA data: the data were downloaded from The Cancer Genome Atlas (TCGA) data portal (https://portal.gdc.cancer.gov/) though R package TCGAbiolinks (https://bioconductor.org/packages/release/bioc/html/TCGAbiolinks.html). Gide cohort data are available from NCBI Sequence Read Archive (SRA) repository under project no. PRJEB23709. Liu cohort data and Miao cohort data are collected from supplementary materials of original article (Liu: https://www.nature.com/articles/s41591-019-0654-5#Sec32; Miao: “https://www.ncbi.nlm.nih.gov/pmc/articles/PMC6035749/”). Mariathasan cohort data are collected from R package “Imvigor_210” (http://research-pub.gene.com/IMvigor210CoreBiologies/). Van Allen cohort data are public in github (https://github.com/vanallenlab/VanAllen_CTLA4_Science_RNASeq_TPM).

## Abbreviations

ACC: Adrenocortical carcinoma
BLCA: Bladder urothelial carcinoma
BRCA: Breast invasive carcinoma
CESC: Cervical squamous cell carcinoma and endocervical adenocarcinoma
CHOL: Cholangiocarcinoma
COAD: Colon adenocarcinoma
DLBC: Lymphoid neoplasm diffuse large B cell lymphoma
ESCA: Esophageal carcinoma
GBM: Glioblastoma multiforme
HNSC: Head and neck squamous cell carcinoma
KICH: Kidney chromophobe
KIRC: Kidney renal clear cell carcinoma
KIRP: Kidney renal papillary cell carcinoma
LAML: Acute myeloid leukemia
LGG: Brain lower grade glioma
LIHC: Liver hepatocellular carcinoma
LUAD: Lung adenocarcinoma
LUSC: Lung squamous cell carcinoma
MESO: Mesothelioma
OV: Ovarian serous cystadenocarcinoma
PAAD: Pancreatic adenocarcinoma
PCPG: Pheochromocytoma and paraganglioma
PRAD: Prostate adenocarcinoma
READ: Rectum adenocarcinoma
SARC: Sarcoma
SKCM: Skin cutaneous melanoma
STAD: Stomach adenocarcinoma
TGCT: Testicular germ cell tumors
THCA: Thyroid carcinoma
THYM: Thymoma
UCEC: Uterine corpus endometrial carcinoma
UCS: Uterine carcinosarcoma
UVM: Uveal melanoma.

## Supplementary Materials

Supplementary Materials Figure S1: enrichment plots of the CD8+ T cell signatures in TCGA. Figure S2: enrichment plots of the dendritic cell signatures in TCGA. Figure S3: enrichment plots of the type 1 helper cell signatures in TCGA. Figure S4: enrichment plots of the Louis IFN-*γ* signatures in TCGA. Figure S5: enrichment plots of the Mark IFN-*γ* signatures in TCGA. Figure S6: enrichment plots of the Padmanee IFN-*γ* signatures in TCGA. Figure S7: survival analysis of PDL2 in TCGA pooled cohort.
