# Supplementary figures and images for: Programmed Cell Death Protein Ligand 2 Is a Potential Biomarker That Predicts the Efficacy of Immunotherapy
Source: Dis Markers. 2021 Oct 31;2021:9453692. doi: 10.1155/2021/9453692 (PMC8572643; doi:10.1155/2021/9453692)

# CD8+ T Cell Signature

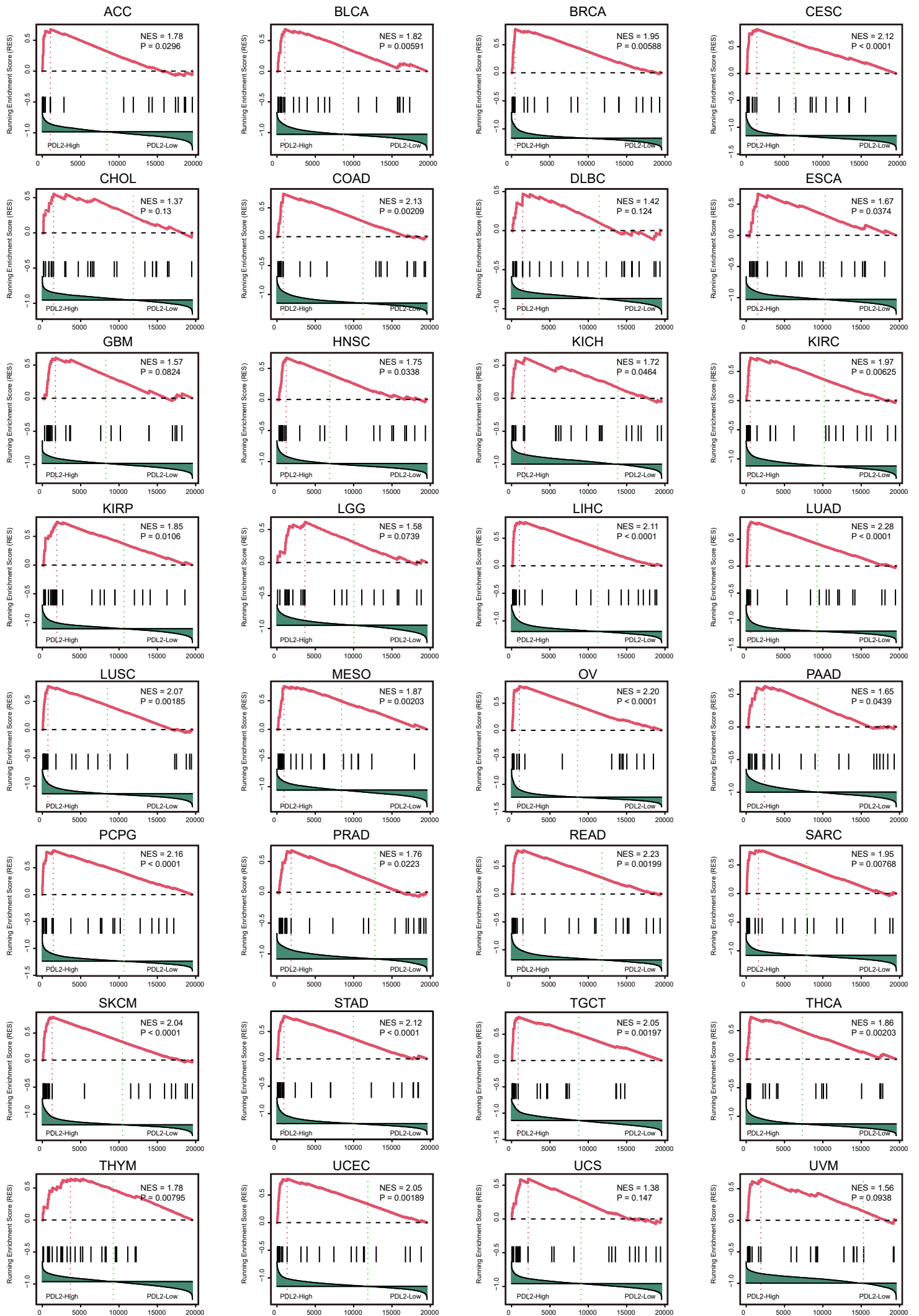

Supplement: Supplementary Materials — Figure S1: enrichment plots of the CD8+ T cell signatures in TCGA. Figure S2: enrichment plots of the dendritic cell signatures in TCGA. Figure S3: enrichment plots of the type 1 helper cell signatures in TCGA. Figure S4: enrichment plots of the Louis IFN-γ signatures in TCGA. Figure S5: enrichment plots of the Mark IFN-γ signatures in TCGA. Figure S6: enrichment plots of the Padmanee IFN-γ signatures in TCGA. Figure S7: survival analysis of PDL2 in TCGA pooled cohort. [file 9453692.f1.zip › Supplementary Fig 1.pdf]

# Dendritic Cell Signature

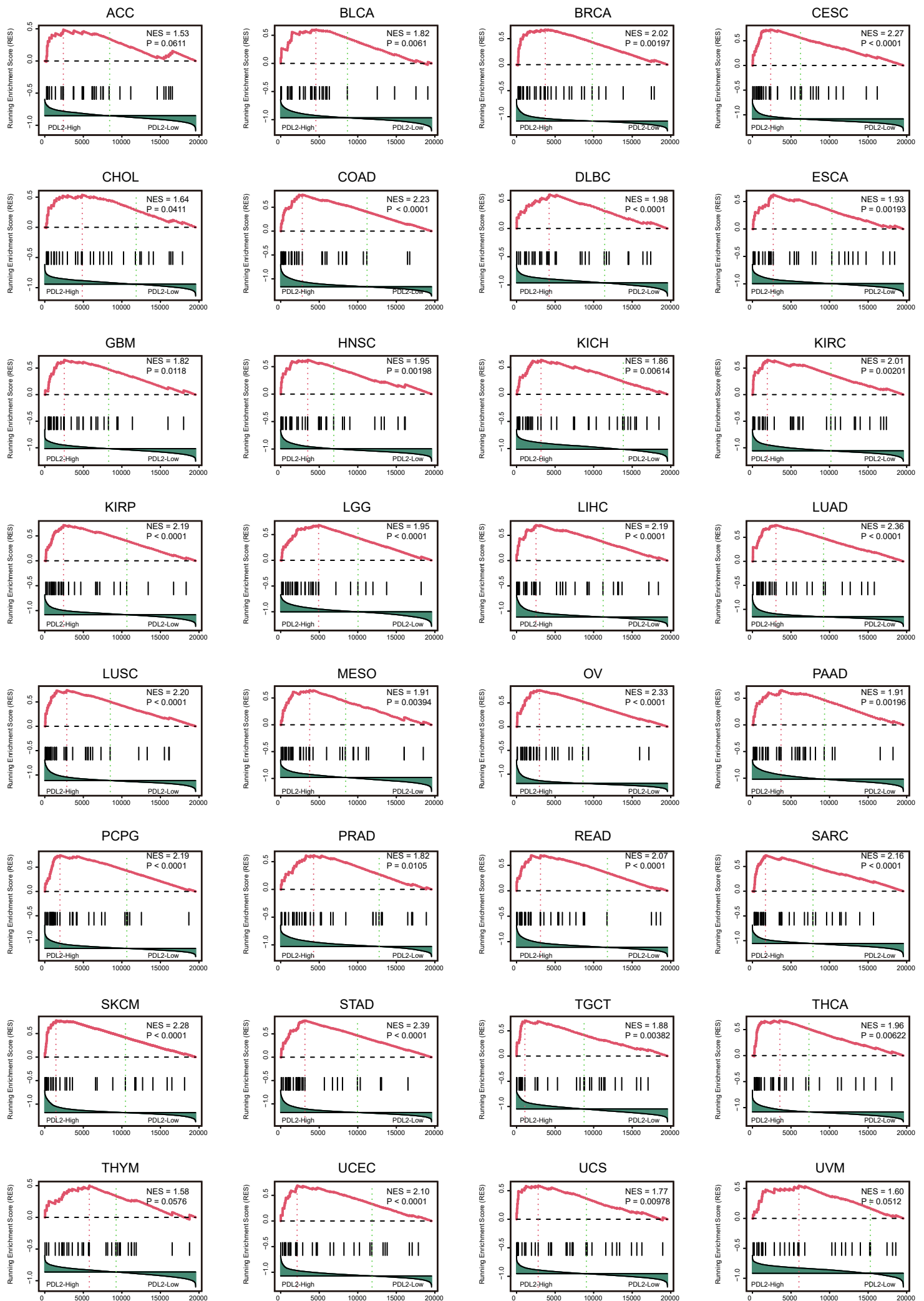

Supplement: Supplementary Materials — Figure S1: enrichment plots of the CD8+ T cell signatures in TCGA. Figure S2: enrichment plots of the dendritic cell signatures in TCGA. Figure S3: enrichment plots of the type 1 helper cell signatures in TCGA. Figure S4: enrichment plots of the Louis IFN-γ signatures in TCGA. Figure S5: enrichment plots of the Mark IFN-γ signatures in TCGA. Figure S6: enrichment plots of the Padmanee IFN-γ signatures in TCGA. Figure S7: survival analysis of PDL2 in TCGA pooled cohort. [file 9453692.f1.zip › Supplementary Fig 2.pdf]

# Type 1 Helper Cell Signature

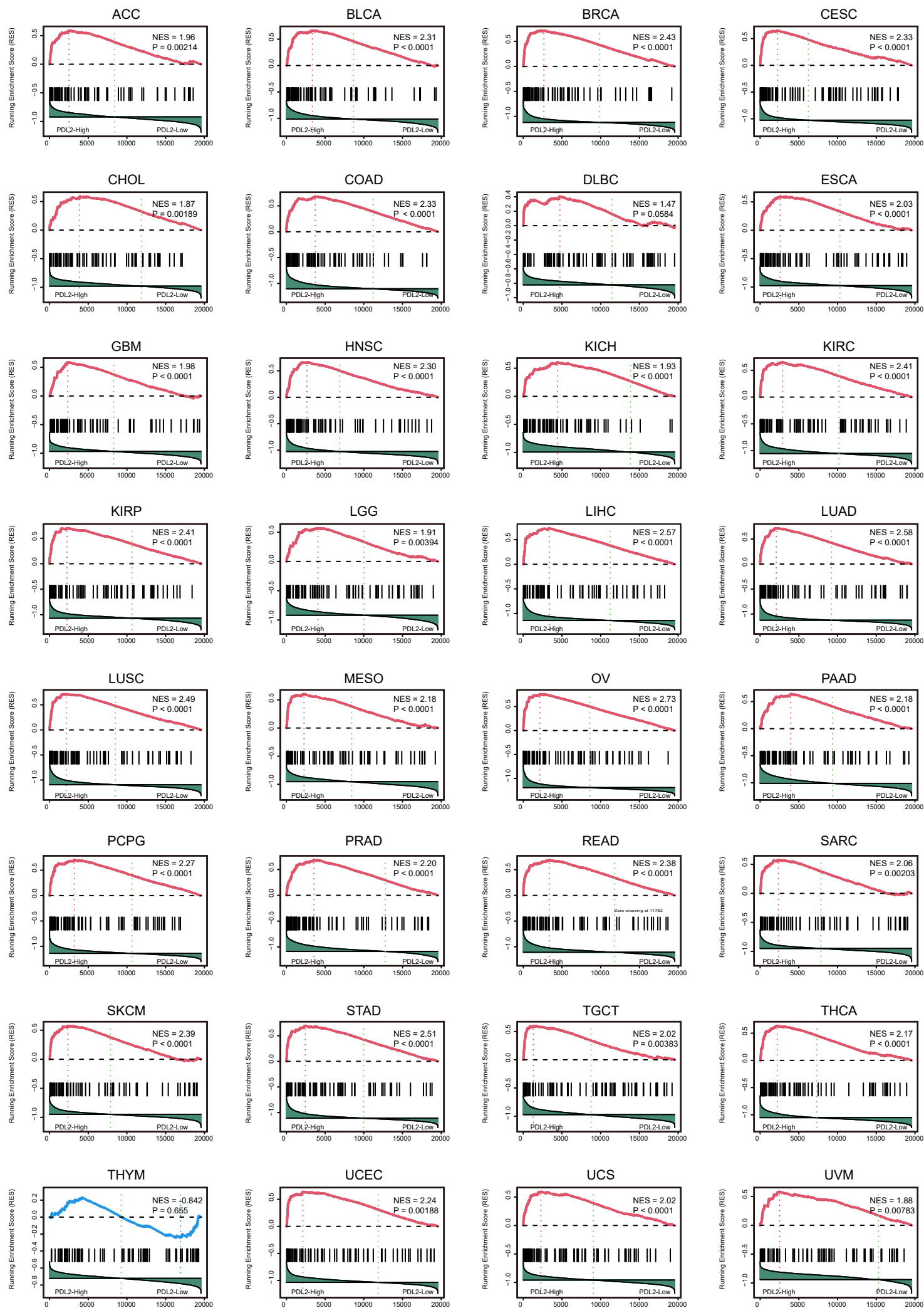

Supplement: Supplementary Materials — Figure S1: enrichment plots of the CD8+ T cell signatures in TCGA. Figure S2: enrichment plots of the dendritic cell signatures in TCGA. Figure S3: enrichment plots of the type 1 helper cell signatures in TCGA. Figure S4: enrichment plots of the Louis IFN-γ signatures in TCGA. Figure S5: enrichment plots of the Mark IFN-γ signatures in TCGA. Figure S6: enrichment plots of the Padmanee IFN-γ signatures in TCGA. Figure S7: survival analysis of PDL2 in TCGA pooled cohort. [file 9453692.f1.zip › Supplementary Fig 3.pdf]

# Louis IFN- $\gamma$ Signature

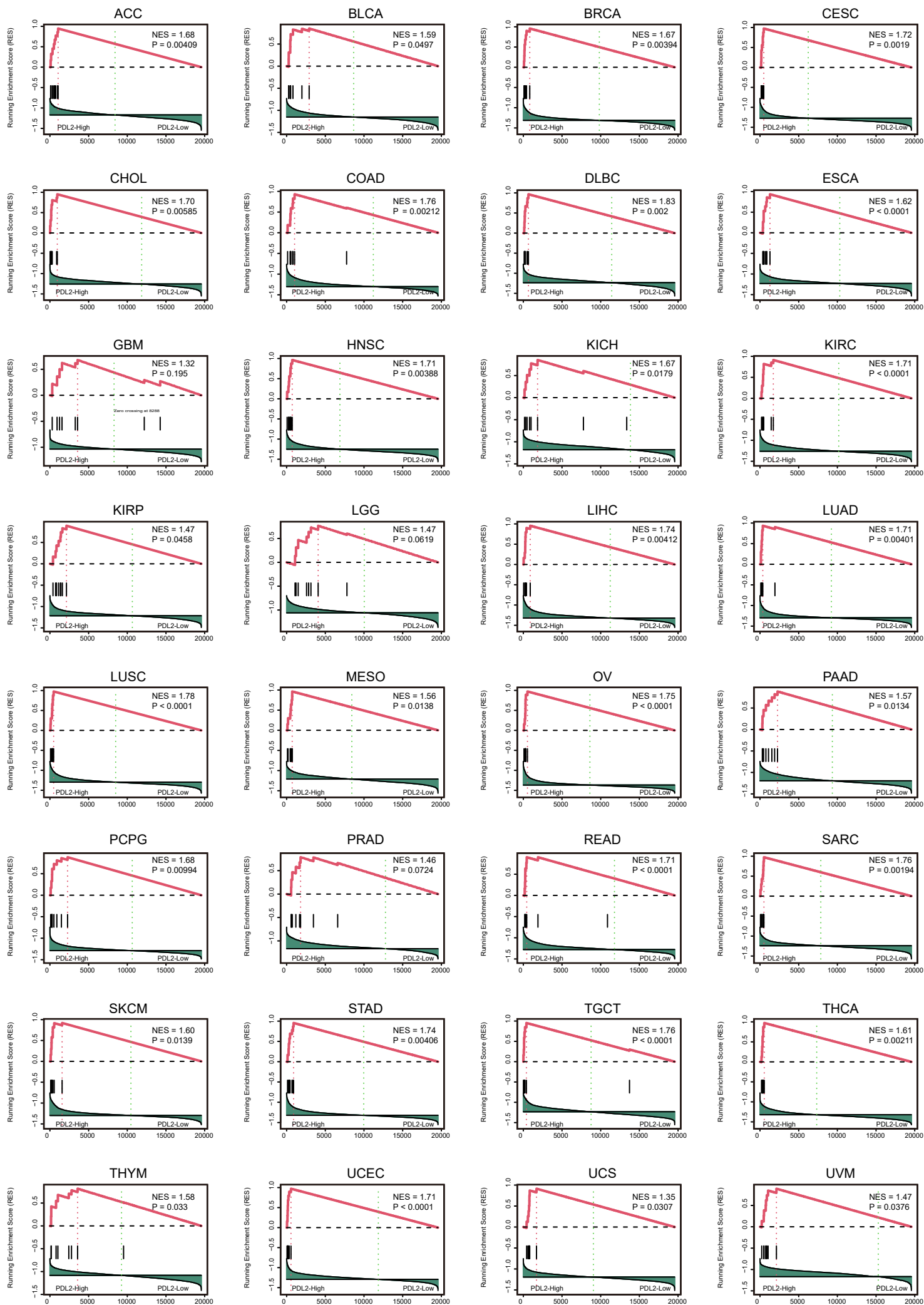

Supplement: Supplementary Materials — Figure S1: enrichment plots of the CD8+ T cell signatures in TCGA. Figure S2: enrichment plots of the dendritic cell signatures in TCGA. Figure S3: enrichment plots of the type 1 helper cell signatures in TCGA. Figure S4: enrichment plots of the Louis IFN-γ signatures in TCGA. Figure S5: enrichment plots of the Mark IFN-γ signatures in TCGA. Figure S6: enrichment plots of the Padmanee IFN-γ signatures in TCGA. Figure S7: survival analysis of PDL2 in TCGA pooled cohort. [file 9453692.f1.zip › Supplementary Fig 4.pdf]

# Mark IFN- $\gamma$ Signature

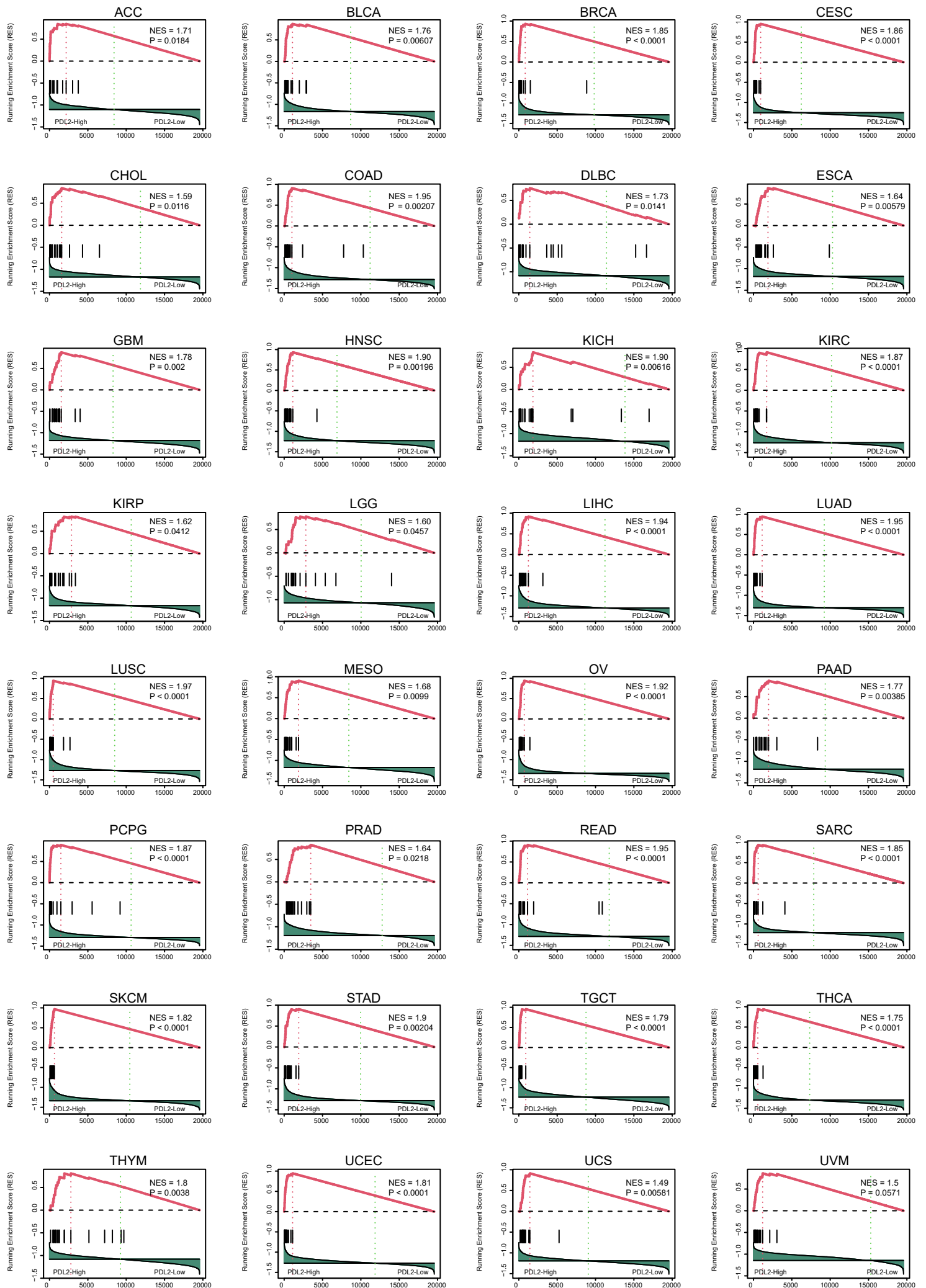

Supplement: Supplementary Materials — Figure S1: enrichment plots of the CD8+ T cell signatures in TCGA. Figure S2: enrichment plots of the dendritic cell signatures in TCGA. Figure S3: enrichment plots of the type 1 helper cell signatures in TCGA. Figure S4: enrichment plots of the Louis IFN-γ signatures in TCGA. Figure S5: enrichment plots of the Mark IFN-γ signatures in TCGA. Figure S6: enrichment plots of the Padmanee IFN-γ signatures in TCGA. Figure S7: survival analysis of PDL2 in TCGA pooled cohort. [file 9453692.f1.zip › Supplementary Fig 5.pdf]

# Padmanee IFN- $\gamma$ Signature

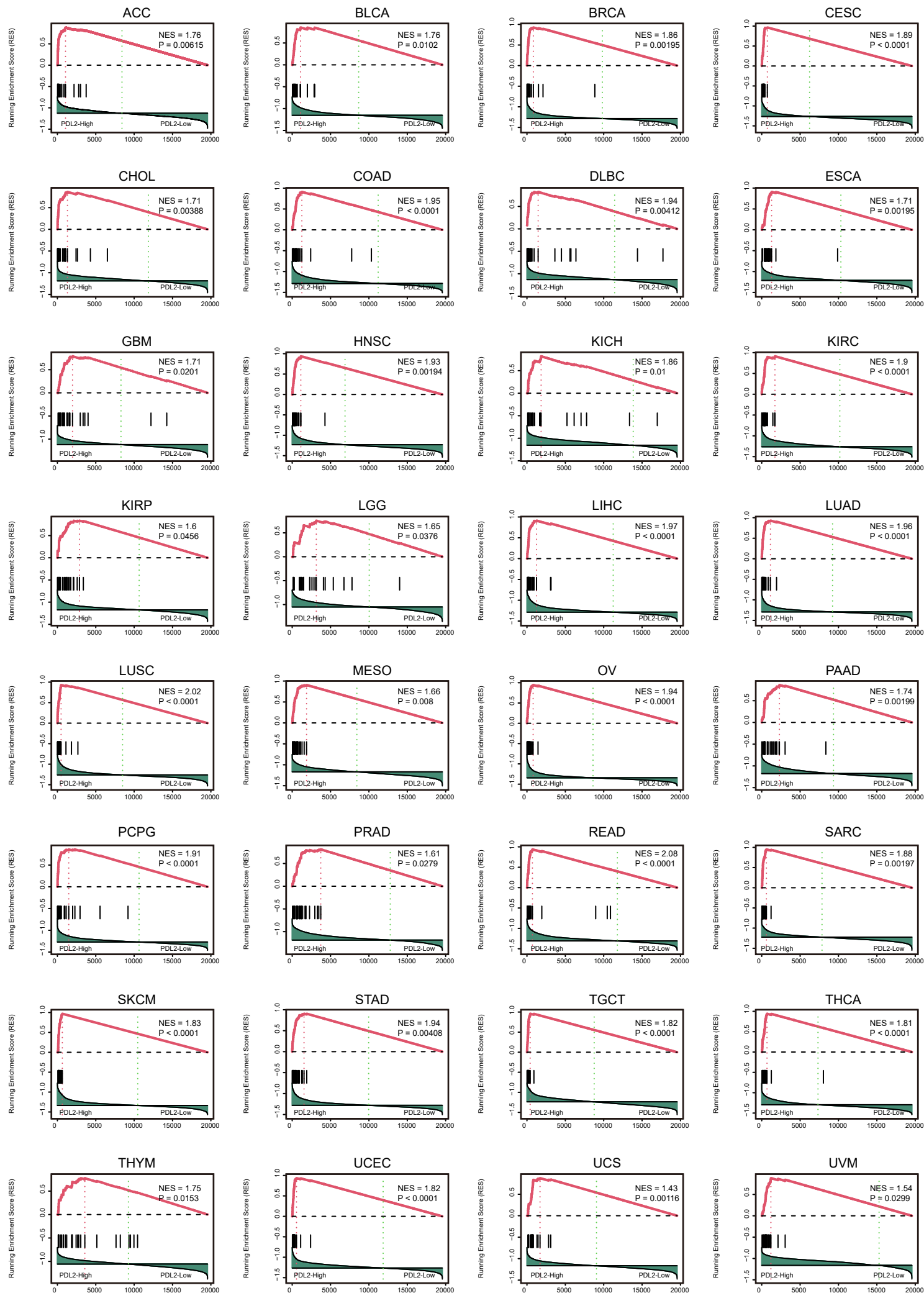

Supplement: Supplementary Materials — Figure S1: enrichment plots of the CD8+ T cell signatures in TCGA. Figure S2: enrichment plots of the dendritic cell signatures in TCGA. Figure S3: enrichment plots of the type 1 helper cell signatures in TCGA. Figure S4: enrichment plots of the Louis IFN-γ signatures in TCGA. Figure S5: enrichment plots of the Mark IFN-γ signatures in TCGA. Figure S6: enrichment plots of the Padmanee IFN-γ signatures in TCGA. Figure S7: survival analysis of PDL2 in TCGA pooled cohort. [file 9453692.f1.zip › Supplementary Fig 6.pdf]

## Survival analysis of PDL2 in TCGA Pooled Cohort

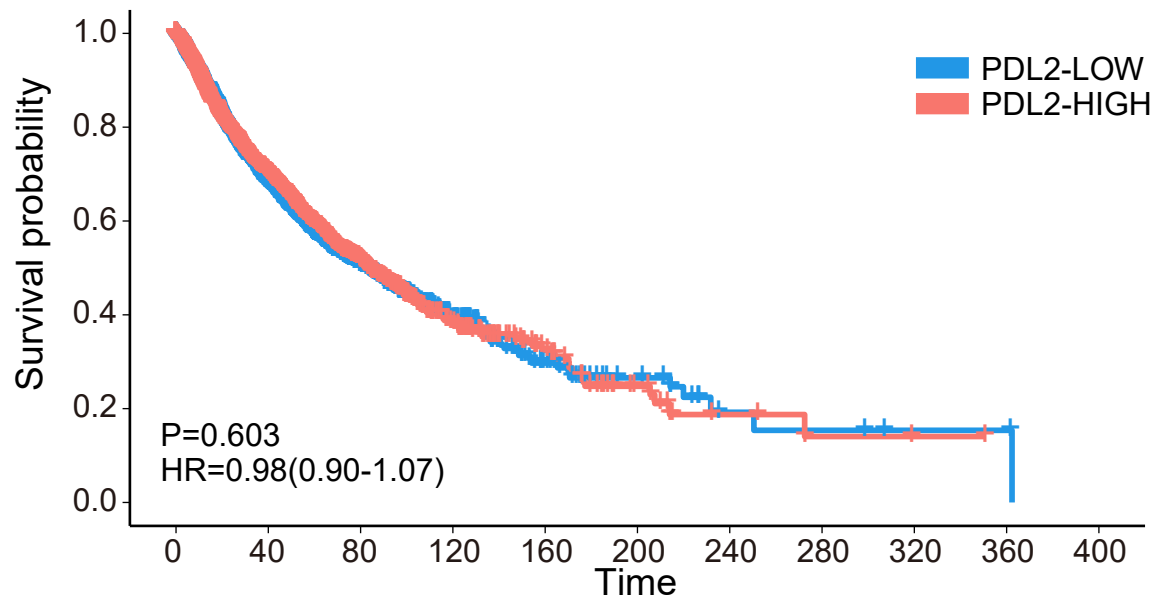

Supplement: Supplementary Materials — Figure S1: enrichment plots of the CD8+ T cell signatures in TCGA. Figure S2: enrichment plots of the dendritic cell signatures in TCGA. Figure S3: enrichment plots of the type 1 helper cell signatures in TCGA. Figure S4: enrichment plots of the Louis IFN-γ signatures in TCGA. Figure S5: enrichment plots of the Mark IFN-γ signatures in TCGA. Figure S6: enrichment plots of the Padmanee IFN-γ signatures in TCGA. Figure S7: survival analysis of PDL2 in TCGA pooled cohort. [file 9453692.f1.zip › Supplementary Fig 7.pdf]
